# O14 An atypical femoral fracture without a history of bisphosphonate use

**DOI:** 10.1093/rap/rkab067.013

**Published:** 2021-10-19

**Authors:** Mark Ford, Srinivasan Venkatachalam

**Affiliations:** The Royal Wolverhampton NHS Trust, Wolverhampton, United Kingdom

## Abstract

**Case report - Introduction:**

A 60-year-old female presented with an insufficiency fracture of the midshaft of the left femur and a persistently low serum alkaline phosphatase. A diagnosis of adult hypophosphatasia was made. We present this case alongside a series of other cases that we have identified locally and discuss the importance of identifying this condition in clinical practice.

**Case report - Case description:**

A 60-year-old female presented with left thigh pain and an 8-year history of polyarthralgia involving the medium-large sized joints. She had a traumatic fracture of the humerus 4 years previously. She had no history or examination findings suggestive of an inflammatory arthritis, connective tissue disease or systemic illness.

An X-ray of left femur revealed a cortical-based lucency at the lateral cortex of midshaft. An MRI identified a focal area of cortical thickening but no evidence of bone marrow oedema suggestive of an osteoid osteoma. Finally, a CT scan confirmed the diagnosis of a healed stress fracture. The patient had never received any form of bisphosphonate treatment, no previous history of a non-traumatic fracture and no history of dental problems.

Laboratory evaluation revealed persistently low levels of serum alkaline phosphatase (∼23 IU/L) with a normal vitamin D and PTH level. Bone density scan showed a T-score of -1.1 at the femoral neck and -2.6 at the lumbar spine. Serum pyridoxal phosphate level was elevated at 457 nmol/L (35-100). A diagnosis of adult hypophosphatasia was suspected and genetic study for ALPL gene confirmed the diagnosis.

The following table summarises a number of other cases of adult hypophosphatasia, which we have identified locally.

**Case report - Discussion:**

Hypophosphatasia (HPP) is a rare inherited metabolic disease caused by mutations in the alkaline phosphatase liver type (ALPL) gene, localized on the chromosome region 1p36.1–34, encoding the tissue-nonspecific isoenzyme of alkaline phosphatase (TNSALP). The disease is related to a decrease in both level and activity of alkaline phosphatase enzyme (ALP). The clinical expression is highly variable, ranging from lethal forms to mild forms diagnosed in adults. Fractures, chondrocalcinosis, calcific periarthritis, and chronic muscle and bone pain are key features for the diagnosis of HPP in adults. Teriparatide has been used for treatment in some case reports with inconsistent results. Antiresorptive drugs are contraindicated as they can worsen underlying osteomalacia.

**Case report - Key learning points:**

Check for persistent low serum alkaline phosphatase levels in adults presenting with fragility fractures, crystal-related arthropathies and nonspecific generalised aches.

Avoid bisphosphonates in patients with low alkaline phosphatase.

**Table:** 

Case	Age / Gender	Presentation	Alkaline phospha tase (IU/L)	Pyridoxal phosphate (nmol/L)	Notes
1 (Index case)	62 / F	Widespread pain, Atypical femoral fracture	23	457	Genetic study confirmed
2	78 / F	Widespread pain, Osteomalacia	22	201	Genetic study confirmed
3	56 / M	Son of Case 2, Osteoporosis, Diabetes mellitus	22 – 29	awaited	Bisphosphonate stopped Genetic study awaited
4	40 / F	Recurrent metatarsal fractures, Low BMD	24	Normal	Bisphosphonate stopped
5	48 / F	Premature loss of teeth, fatigue, Widespread pain, Diabetes mellitus, hyperlipidaemia	13-23	327	Genetic study confirmed
6	75 / F	Seronegative polyarthritis	21-26	120	Genetic study awaited

